# Effects of 3-month CPAP therapy on brain structure in obstructive sleep apnea: A diffusion tensor imaging study

**DOI:** 10.3389/fneur.2022.913193

**Published:** 2022-08-22

**Authors:** Xiang Liu, Zhipeng Wei, Liting Chen, Wenfeng Duan, Haijun Li, Linghong Kong, Yongqiang Shu, Panmei Li, Kunyao Li, Wei Xie, Yaping Zeng, Ling Huang, Ting Long, Dechang Peng

**Affiliations:** ^1^Department of Radiology, The First Affiliated Hospital of Nanchang University, Nanchang, China; ^2^Department of Radiology, The Second Affiliated Hospital of Nanchang University, Nanchang, China; ^3^Medical Imaging Center, First Affiliated Hospital of Jinan University, Guangzhou, China

**Keywords:** CPAP, diffusion tensor imaging, white matter fibers, TBSS, cognitive impairment

## Abstract

White matter (WM) fiber alterations in patients with obstructive sleep apnea (OSA) is associated with cognitive impairment, which can be alleviated by continuous positive airway pressure (CPAP). In this study, we aimed to investigate the changes in WM in patients with OSA at baseline (pre-CPAP) and 3 months after CPAP adherence treatment (post-CPAP), and to provide a basis for understanding the reversible changes after WM alteration in this disease. Magnetic resonance imaging (MRI) was performed on 20 severely untreated patients with OSA and 20 good sleepers. Tract-based spatial statistics was used to evaluate the fractional anisotropy (FA), mean diffusion coefficient, axial diffusion coefficient, and radial diffusion coefficient (RD) of WM. To assess the efficacy of treatment, 20 patients with pre-CPAP OSA underwent MRI again 3 months later. A correlation analysis was conducted to evaluate the relationship between WM injury and clinical evaluation. Compared with good sleepers, patients with OSA had decreased FA and increased RD in the anterior thalamic radiation, forceps major, inferior fronto-occipital tract, inferior longitudinal tract, and superior longitudinal tract, and decreased FA in the uncinate fasciculus, corticospinal tract, and cingulate gyrus (*P* < 0.05). No significant change in WM in patients with post-CPAP OSA compared with those with pre-CPAP OSA. Abnormal changes in WM in untreated patients with OSA were associated with oxygen saturation, Montreal cognitive score, and the apnea hypoventilation index. WM fiber was extensively alteration in patients with severe OSA, which is associated with cognitive impairment. Meanwhile, cognitive recovery was not accompanied by reversible changes in WM microstructure after short-term CPAP therapy.

## Introduction

Obstructive sleep apnea (OSA) is a common sleep disorder that is characterized by repeated partial collapse and obstruction of the upper respiratory tract, resulting in intermittent hypoxia, hypercapnia, and sleep fragmentation (1). Chronic intermittent hypoxemia and sleep fragmentation lead to a cascade of pathophysiological processes, including oxidative stress, systemic inflammation, neuroinflammation, and neuronal death (2, 3). Hypoxemia, inflammation, and oxidative stress are widely recognized to be associated with cognitive impairment in patients with OSA (4–6). Although clinical and pathophysiological support for OSA may lead to damage to white matter (WM) structures, the exact neural mechanisms underlying cognitive impairment in patients with OSA are complex and unclear.

Continuous positive airway pressure (CPAP) is currently considered the first-line treatment for OSA (7). CPAP therapy can significantly improve daytime sleepiness in patients with OSA and partially recover their cognitive dysfunction (8). The efficacy of CPAP therapy in asymptomatic, mild, and severe patients with OSA has not been fully established, particularly in the recovery of cognitive dysfunction (9). Therefore, determining whether OSA treatment is stable and whether brain function and structure are reversed has become a hot topic.

Neuroimaging methods can explore brain changes after CPAP therapy and provide new insights into the recovery from cognitive dysfunction in patients with OSA after treatment. A study on voxel-based morphometry showed increased hippocampal and frontal gray matter (GM) volume and no significant increase in total intracranial GM volume after 3 months of CPAP therapy, whereas another study found no change in GM volume after 6 months of CPAP therapy (10). Concerns have been raised regarding the factors that might affect the effectiveness of CPAP therapy. Castronovo et al. demonstrated that WM fiber is changed in patients with OSA in the baseline group. Moreover, they reported that fractional anisotropy (FA) and mean diffusion coefficient (MD) in some brain regions can be recovered after 12 months of CPAP therapy and are also associated with attention, executive performance, and short-term memory recovery. This confirmed the reversible WM structural changes in patients with OSA after long-term CPAP therapy (11). Damage to the WM myelin sheath is associated with persistent daytime sleepiness, which was also demonstrated in another CPAP study (12). Our previous study showed that after 1 month of CPAP treatment, spontaneous brain activity in the bilateral posterior cerebellar lobe, right superior temporal gyrus, left anterior central gyrus, and other brain regions was reversed, which was related to cognitive function and drowsiness. Such reversible spontaneous brain activity is related to the adaptive compensation of OSA (13). However, few studies were conducted on whether WM structure is reversed after CPAP therapy.

Diffusion tensor imaging (DTI) is a new imaging method based on diffusion-weighted imaging that can describe changes in brain structure (14). DTI can analyze the dispersion movement of water molecules in tissues in three-dimensional space and non-invasively observe the changes in WM structure and fiber bundles in the brain (15), which is used to study the pathophysiological changes in the early brain (16). Based on the advantages of voxel and trajectory analysis, tract-based spatial statistics (TBSS) has been widely applied in studies of various neurological diseases, such as Parkinson's disease (17), chronic spinal cord injury in children (18), multiple sclerosis (19), schizophrenia (20), and preoperative evaluation of tumor (21). Macey et al. (22) showed that OSA is more likely to damage WM fibers (bilateral cingulate, right terminal striatum) in women, and this differential damage helps explain the sex differences in symptoms, particularly anxiety and depression. Kumar et al. (23) reported that the MD values of multiple brain regions in patients with OSA are significantly reduced, which is related to the pathological mechanism of ischemia and hypoxia induction. We believe that TBSS can provide new insights into OSA-related WM fiber damage and its underlying neuropathological mechanisms.

In this study, we aimed to evaluate the changes in WM microstructure in patients with OSA after short-term treatment and explore the correlation between the partially reversed areas of WM and the recovery of neurocognitive function in patients with OSA.

## Materials and methods

### Patients

**Figure 1:**
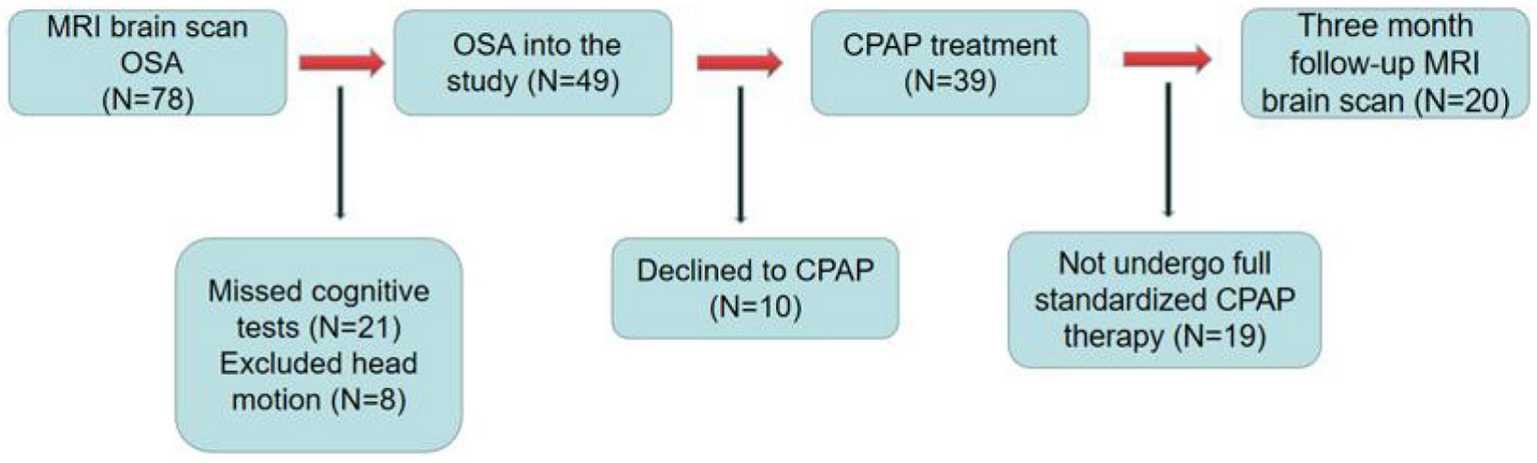
Schematic diagram of OSA patients process in this study.

This was a short-term, longitudinal study. Figure 1 shows the recruitment of patients with OSA. The sleep health group was assessed only at baseline. Good sleepers (GSs) were recruited through community advertisement. All patients with OSA were enrolled in the Sleep Monitoring Center, Department of Respiratory Medicine, The First Affiliated Hospital of Nanchang University, from August 2020 to March 2022. The diagnostic criteria were the 2017 American Academy of Sleep Medicine Clinical Practice Guidelines for obstructive respiratory sleep apnea in adults (24). The inclusion criteria were as follows: apnea hypoventilation index (AHI) >15, age 20-60 years, right-handedness, and standard CPAP therapy for 3 months, 4 h per night, at least 5 days per week. The exclusion criteria for all participants were as follows: (1) sleep disorders other than OSA; (2) respiratory disease, hypertension, diabetes, hypothyroidism, and prior CPAP treatment; (3) history of central nervous system diseases, including neurodegenerative diseases, epilepsy, brain tumors, cerebral infarction, and traumatic brain injury; (4) abuse of illegal drugs or current use of psychoactive drugs; (5) MRI contraindications, such as internal metal implants and claustrophobia; (6) motion artifacts; and (7) loss to follow-up. Finally, 20 GSs, and 20 patients with OSA who had complied with CPAP therapy for 3 months were included in the analysis. We adhered to the principles of the Declaration of Helsinki. This study was approved by the Medical Ethics Committee of the First Affiliated Hospital of Nanchang University [2020 (94)]. All participants provided written informed consent.

### Neuropsychological assessment and polysomnography

All patients with OSA completed neuropsychological assessments, including the Montreal cognitive assessment (MoCA), Epworth sleepiness scale (ESS), and Pittsburgh sleep quality index, at baseline and at 3-month follow-up. MoCA includes visual space and execution, naming, memory, attention, language, abstract thinking, computation, and direction (25). The highest MoCA score was 30, with a score of <26 indicating the presence of cognitive impairment. If the number of years of education was <12, one point was added to the total score to adjust the education level. The ESS contains eight different categories used to assess daytime sleepiness in patients with OSA. The overall ESS score was 24 points (3 points for each category), with 0, 6, 11, and 16 corresponding to four different levels of sleepiness (26). The PSQI is used to assess sleep quality on a scale of 0–21, with higher scores indicating poorer sleep quality (27). The Hamilton anxiety scale (HAMA) and Hamilton depression scale were used to assess anxiety and depression, respectively. In general, a HAMA score of >14 indicates clinically significant anxiety symptoms. A HAMD score of <7 is normal; a score of 7–17, possible depression; an overall score of 17–24, definite depression; and a total score of >24, major depression.

To rule out sleep disorders other than OSA, all subjects with OSA and GSs were required to undergo polysomnography (PSG) with a physiological monitoring system (Alice 5 LE; Respironics, Orlando, FL, USA), the detection contents included electroencephalogram, electroophthalmogram, electrocardiogram, oxygen saturation (SaO_2_), etc. The day before polysomnography, all participants avoided hypnotics, alcohol, and coffee, and the test was performed from 10 p.m. to 6 a.m. the following day. According to the American Academy of Sleep Medicine's clinical practice guidelines for adult OSA, an AHI between 5 and 15 is classified as mild; AHI ≥15/h but <30/h, moderate; and AHI ≥30/h, severe (28).

### CPAP treatment

An automatic CPAP equipment (YH-480; Yuwell, Jiangsu, China) was used to explain the basic functions of the CPAP machine operation in patients with OSA who met the inclusion criteria, and an appropriate mask interface was selected. The equipment can monitor breathing levels and automatically adjust the pressure. The treatment pressure of the ventilator was set to 4–20 cm H_2_O. All subjects with OSA received standard CPAP therapy for 3 months with compliance of ≥4 h per night for at least 5 days per week. The built-in SIM card of the ventilator can upload user data in real time *via* a wireless transmission function, including the usage duration, AHI, and mask leakage.

### MRI data acquisition

All participants were scanned on a 3.0 Tesla MRI system with an eight-channel phased-array head coil (Siemens, Munich, Germany) between 7 and 9 p.m. The scans included conventional MRI and DTI of the head. During the examination, subjects were required to lie quietly on the examination bed, wear ear plugs and eye masks throughout the examination to reduce external interference, fix their heads with sponges to prevent head movement, and breathe through the nose to minimize the interference of head artifacts caused by mouth opening breathing. Routine MRI data were collected from the brain: axis T2WI [pulse repetition time (TR) = 3,000 ms, echo time (TE) = 122 ms, field of view (FOV) = 240 mm × 240 mm, matrix = 256 × 256, layer thickness = 5 mm], axial T1WI (TR = 600 ms, TE = 10 ms, FOV = 240 mm × 240 mm, matrix = 256 × 256, layer thickness = 5 mm), and axial T2-FLAIR (TR = 3,000 ms, TE = 122 ms, FOV = 240 mm × 240 mm, matrix = 256 × 256, layer thickness = 5 mm). Conventional craniocerebral MRI was used to exclude cerebral parenchymal structural lesions such as cerebral infarction, large cysts, and tumors, and was performed by two imaging physicians with senior professional titles. Subsequently, DTI was performed: single-shot spin echo-echo planar image of pulse spin-echo diffusion-weighted image sequence of subjects was obtained, and its parameters were as follows: TR = 7,700 ms, TE = 104 ms, FOV = 230 × 230 mm, voxel size = 1.8 × 1.8 × 2.0 mm, matrix = 128 × 128, excitation number = 2, and layer thickness = 2.0 mm. Sixty-four non-linear diffusion gradient directions were applied with b values of 0 and 1,000 s/mm^2^.

### Data preprocessing

All DTI data were preprocessed using FSL V5.0.9 (FMRIB Software Library, http://www.fmri.ox.au.uk/fsl) developed by the Functional Magnetic resonance Imaging Center of the University of Oxford (29), and TBSS was used for voxel statistical analysis (30). The following preprocessing steps were performed. First, the format of the original DTI data was converted (from DICOM to NIFTI format), and the data quality of the generated files was checked layer by layer to evaluate the basic parameters of the data, including image artifacts, image layers, and data head movement. Second, head eddy current correction and gradient direction correction were performed to align the data generated in the previous step with image B = 0, and the corresponding gradient direction correction was performed on the original dispersion gradient table. Third, to strip and remove images outside the brain, the threshold was set to 0.2(BET tool using FSL software). Finally, the DTI tensor index was calculated; FA, MD, axial diffusion coefficient (AD), and radial diffusion coefficient (RD) were generated.

DTI data processing was based on TBSS: (1) All FA values were registered in the standard MNI space using non-linear registration parameters. The FMRIB58_FA_1 mm template of the FSL was used as the registration image. (2) The average FA value images of all the test data were generated, and the WM fiber skeleton was then extracted, and the WM fiber skeleton was constructed based on the self-generated data. (3) The FA images formed after previous registration were projected onto the WM fiber skeleton constructed by the data in this study to generate individual FA maps for further statistical analysis. The average FA threshold was 0.2. (4) A design comparison matrix was created, and a randomization tool was used to perform a permutation test on the two groups of FA image data. We set the number of permutations to 5,000, with multiple comparisons correction using threshold-free cluster enhancement (to control type I errors). (5) The differential WM area of the FA map was extracted (*p* < 0.05). The location of the differential WM area could be checked using the DTI-81 WM template of the International Consortium for Brain Mapping (31); (6) Similarly, MD, AD, and RD values were calculated using the NON-FA DTI index based on the FA skeleton foundation, and subsequent statistical analysis was conducted.

### Statistic analysis

Statistical analysis was performed using IBM SPSS Statistics for Windows (version 23.0), and statistical significance was set at *P* < 0.05. Demographic data and clinical psychological scores are expressed as the mean ± standard deviation. The Kolmogorov–Smirnov test was used to determine whether the data conformed to a normal distribution. To compare the differences in clinical indicators between OSA and GSs, pre-CPAP, and post-CPAP, a two-sample *t* test was used for normally distributed data, and the Mann–Whitney U test was used for non-normally distributed data. Spearman correlation analysis was conducted between FA, MD, AD, and RD values of patients with OSA with abnormal brain regions and the clinical evaluation results.

## Results

### Demographic, clinical, and neurocognitive scales

Detailed demographic and clinical assessments of the two groups are shown in Table 1. No significant difference was found in age and years of education between the OSA and GSs groups. However, significant differences were noted in body mass index, AHI, minimum blood oxygen saturation, mean blood oxygen saturation, SaO_2_ <90%, ESS, and MoCA between the two groups. Demographic and clinical evaluations of pre- and post-CPAP are shown in Table 2. Significant differences were found in ESS, MoCA, HAMA, Hamilton depression scale, Pittsburgh sleep quality index, and other aspects between pre- and post-CPAP.

**Table 1:** Demographic and clinical data of OSA and GSs.

**Category**	**OSA**	**GSs**	***t*-**	***P*-**
	**(*n* = 20)**	**(*n* = 20)**	**value**	**value**
Gender (male/female)	19/1	19/1	n/a	n/a
Age (year)^a^	40.81 ± 8.61	39.75 ± 12.36	−0.312	0.855
BMI (Kg/m^2^)^b^	27.12 ± 3.98	23.50 ± 1.90	−3.662	0.001
Education (year)^b^	11.60 ± 2.56	10.75 ± 2.82	−0.996	0.325
AHI, /hour^b^	46.80 ± 19.69	2.54 ± 1.24	−10.03	<0.001
LSaO_2_ (%)^b^	71.89 ± 10.27	93.80 ± 3.28	9.078	<0.001
MSaO_2_ (%)^b^	94.05 ± 3.08	97.25 ± 1.94	3.920	<0.001
SaO_2_ <90%^b^	32.33 ± 15.87	0.25 ± 0.18	−9.037	<0.001
MoCA^b^	23.25 ± 2.57	28.25 ± 1.20	7.867	0.001
ESS^b^	10.40 ± 5.13	3.15 ± 2.13	−5.832	<0.001

*GSs, normal sleep group; AHI, apnea hypopnea index; LSaO2, minimum blood oxygen saturation; MSaO2, average blood oxygen saturation; SaO2 <90%, percentage of total sleep time with oxygen saturation <90; ESS, Epworth sleepiness scale; ^a^, Student, t-test; ^b^, Mann-Whitney U-test. n/a indicates the no statistics*.

**Table 2:** Demographic and clinical data of pre-CPAP and post-CPAP.

**Category**	**pre-CPAP**	**post-CPAP**	***t*-**	***P*-**
	**(*n* = 20)**	**(*n* = 20)**	**value**	**value**
Gender (male/female)	19/1	19/1	n/a	n/a
Age (year)^a^	40.8 ± 8.61	n/a	n/a	n/a
BMI (Kg/m^2^)^a^	27.12 ± 3.98	27.01 ± 3.91	−0.82	0.805
Education (year)^b^	10.75 ± 2.82	n/a	n/a	n/a
AHI, /hour^a^	46.80 ± 19.69	n/a	n/a	n/a
LSaO_2_ (%)^a^	71.89 ± 10.27	n/a	n/a	n/a
MSaO_2_ (%)^a^	94 ± 3.08	n/a	n/a	n/a
SaO2 <90%^a^	32.33 ± 15.87	n/a	n/a	n/a
MoCA^a^	23.25 ± 2.57	25.65 ± 2.70	2.87	0.007
HAMA^b^	7.30 ± 5.11	3.60 ± 2.47	−2.91	0.006
HAMD^b^	4.85 ± 2.56	3.15 ± 2.15	−2.27	0.029
PSQI^b^	6.25 ± 1.80	5.1 ± 1.58	−2.142	0.039
ESS^a^	10.4 ± 5.13	7.15 ± 3.34	−3.372	0.023

*AHI, apnea hypopnea index; LSaO_2_, minimum blood oxygen saturation; MSaO_2_, average blood oxygen saturation; SaO_2_ <90%, percentage of total sleep time with oxygen saturation <90; ESS, Epworth sleepiness scale; HAMA, Hamilton anxiety scale; HAMD, Hamilton depression scale; PSQI, Pittsburgh sleep quality index. ^a^, Student, t-test; ^b^, Mann-Whitney U-test. n/a indicates the no statistics*.

### TBSS

**Figure 2:**
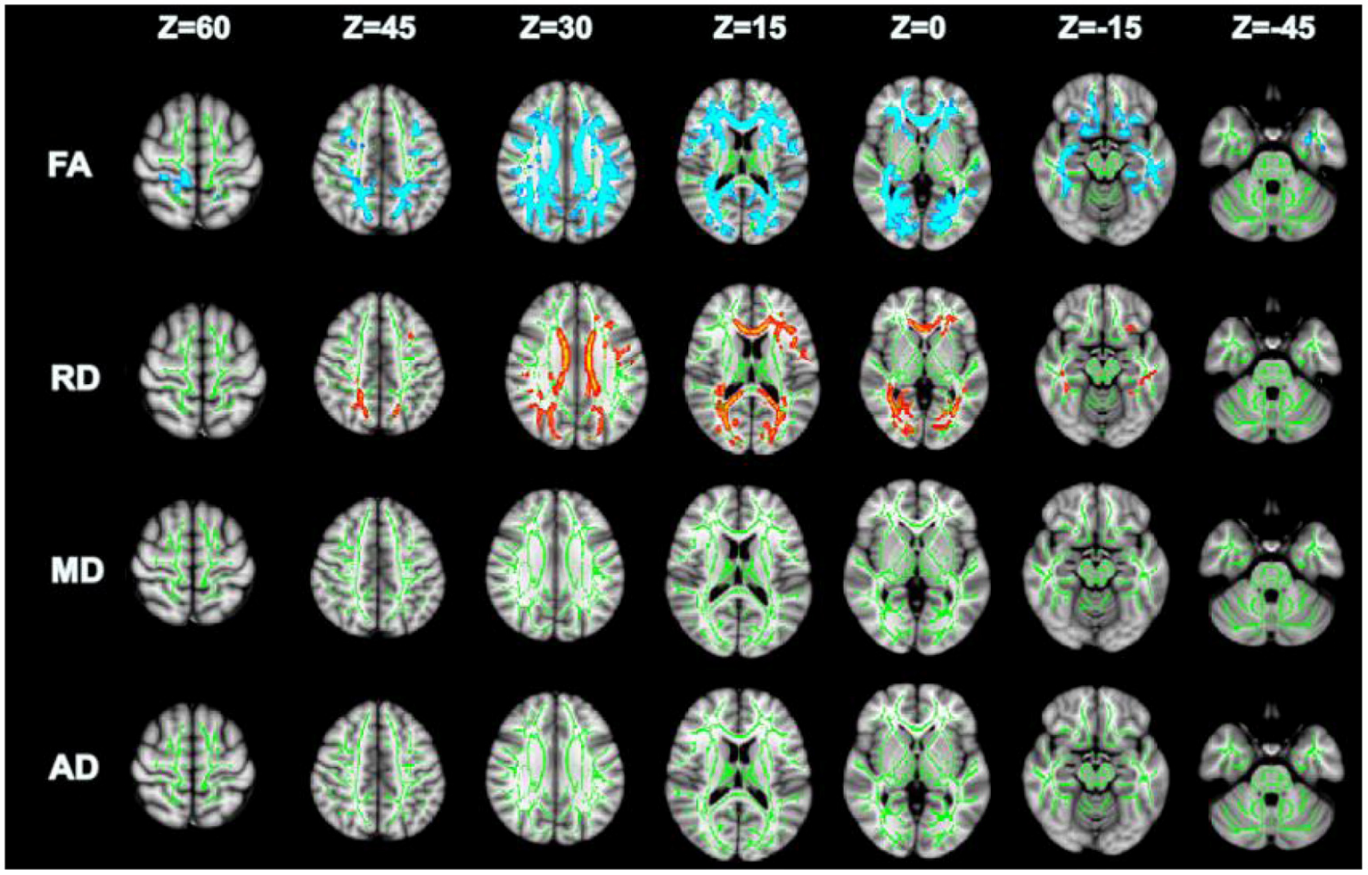
TBSS analysis of OSA and GSs differential white matter fibers, green: mean fractional anisotropy (FA) skeleton; Blue and red represent changed areas (FA and RD) of DTI value in OSA group compared with control group, *P* < 0.05.

**Figure 3:**
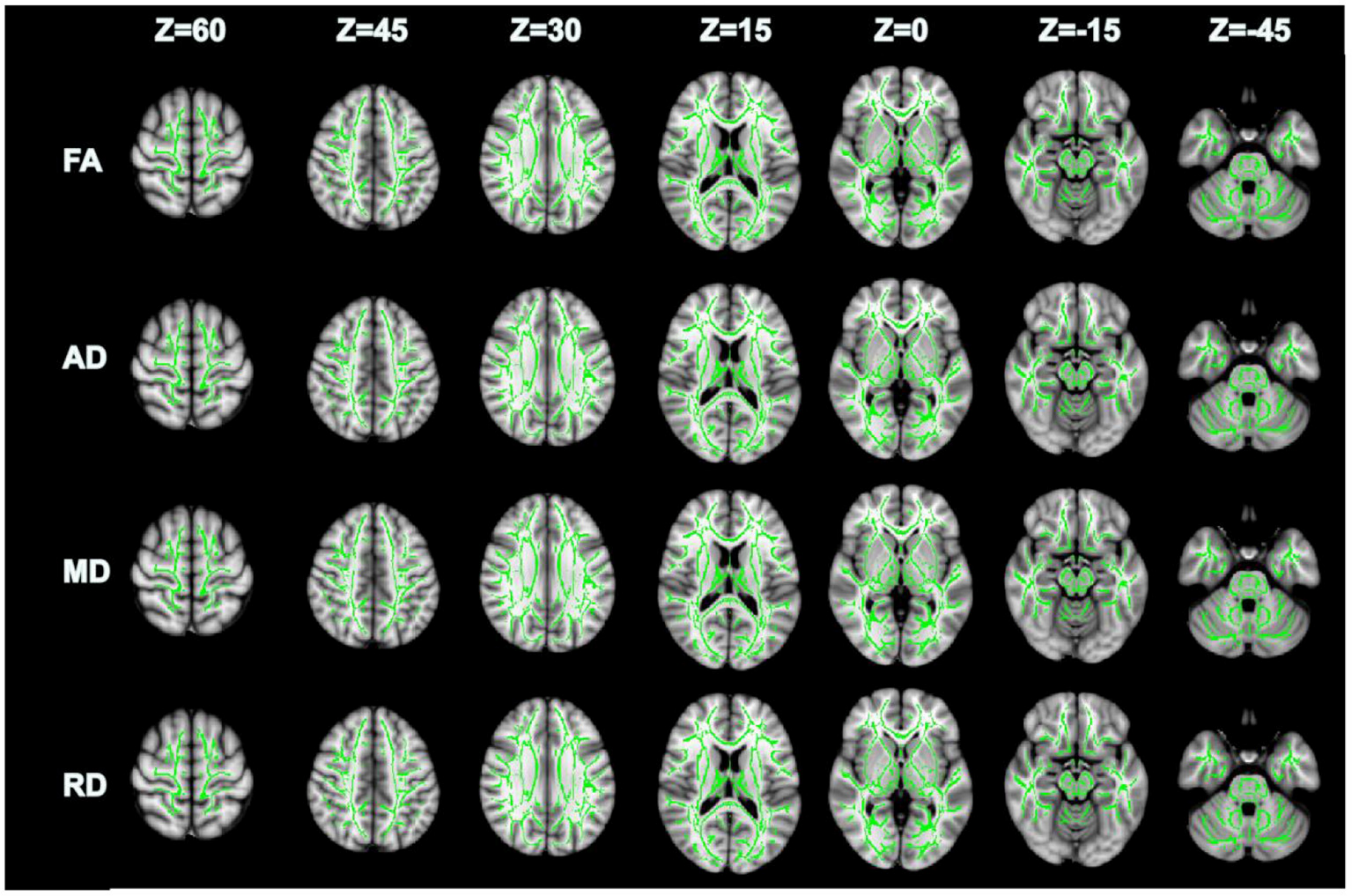
There were no significant differences in FA, AD, MD and RD between pre-CPAP and post-CPAP after TBSS analysis, *P* > 0.05.

Compared with GSs, FA and RD were significantly different in the OSA group (*P* < 0.05; Figure 2). In the OSA group, the FA values of the bilateral anterior thalamic radiation (ATR), corticospinal tract (CST), inferior fronto-occipital tract (IFO), forceps major (FM), superior longitudinal fasciculus (SLF), inferior longitudinal fasciculus (ILF), uncinate fasciculus (UNC), and right cingulate gyrus (CGC) decreased (Table 3). In the OSA group, ATR on the left side, and RD values of the bilateral IFO, FM, ILF, and SLF increased (Table 4). No significant differences were found in FA, AD, RD, and MD between pre- and post-CPAP WM fibers (*P* > 0.05; Figure 3).

**Table 3:** Distribution of FA differences in brain regions between OSA and GSs before treatment.

**Base line**	**Cluster**	**Voxel**	***P*-value**	**MNI**			**L/R**	**WM**
				**X**	**Y**	**Z**		
	15	32,990	0.001	−2	−29	18	L	ATR, FM, IFO, SLF
	14	5,656	0.013	30	19	11	R	ATR, UNC, SLF, IFO
	13	1,482	0.034	−44	−27	−14	L	ILF, SLF, UNC
	12	382	0.036	11	−35	62	R	CST
	11	171	0.037	21	18	−20	R	UNC, IFO
	10	127	0.039	−33	−46	10	L	ATR, IFO, ILF, SLF
	9	123	0.039	−28	−35	19	L	ATR, SLF
	8	66	0.040	25	−90	−1	R	FM, IFO, ILF
	7	54	0.040	30	−4	−14	R	IFO, ILF, UNC
	6	40	0.040	−28	−10	25	L	CST, SLF
	5	39	0.040	10	−51	17	R	CGC

*ATR, Anterior thalamic radiation; CST, Corticospinal tract; FM, Forceps major; IFO, Inferior fronto-occipital fasciculus; ILF, Inferior longitudinal fasciculus; UNC, Uncinate fasciculus; SLF, Superior longitudinal fasciculus; CGC, Cingulate gyrus*.

**Table 4:** Distribution of RD differences in brain regions between OSA and GSs before treatment.

**Base line**	**Cluster**	**Voxel**	***P*-value**	**MNI**			**L/R**	**WM**
				**X**	**Y**	**Z**		
	9	17,745	0.006	−5	−27	21	L	ATR, FM, IFO, ILF, SLF
	8	2,579	0.028	28	−54	6	R	FM, IFO, ILF, SLF
	7	237	0.045	38	−35	−20	L	ILF
	6	171	0.046	33	−79	−4	L	IFO, ILF
	5	90	0.047	−34	−49	11	L	ATR, IFO, ILF, SLF
	4	75	0.047	−54	−26	−13	L	ILF, SLF
	3	73	0.047	−55	−40	−5	L	SLF

*ATR, Anterior thalamic radiation; FM, Forceps major; IFO, Inferior fronto-occipital fasciculus; ILF, Inferior longitudinal fasciculus; SLF, Superior longitudinal fasciculus*.

### Relationship between DTI changes and clinical evaluation in patients with OSA

**Figure 4:**
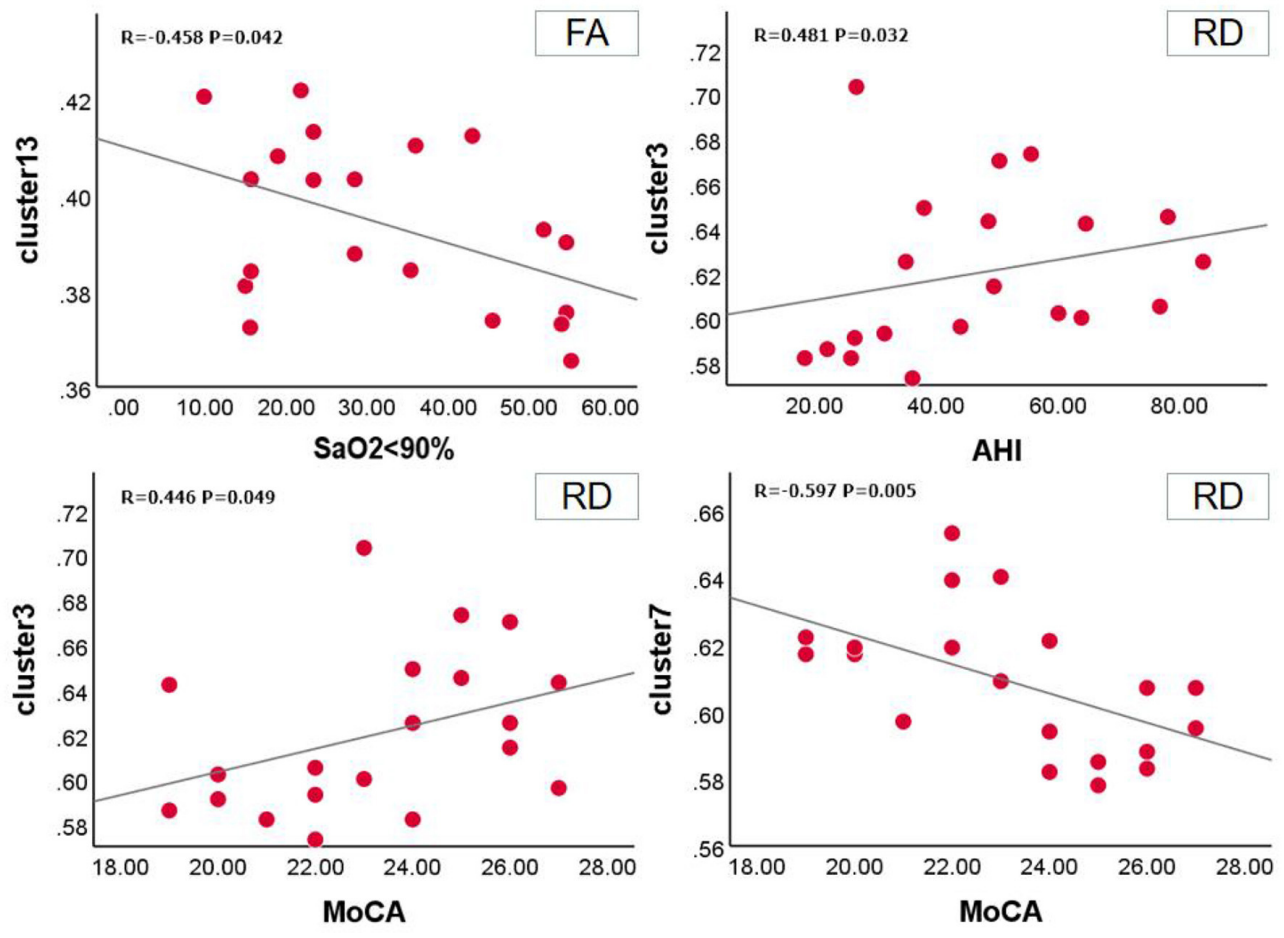
Correlation between FA, RD and clinical assessment scale in OSA before the treatment.

Relationship between FA value and clinical scale in OSA group at baseline. SaO_2_ <90% was positively correlated with clusters 13 (*r* = −0.458, *P* = 0.042, 95% CI: −0.787,−0.025). In the RD value of the OSA group, the MoCA scale was positively correlated with cluster 3 (*r* = 0.446, *P* = 0.049, 95%CI: 0.028, 0.718) and negatively correlated with cluster 7 (*r* = −0.597, *P* = 0.005, 95%CI: −0.724,−0.339), AHI was positively correlated with clusters 3 (*r* = 0.481, *P* = 0.032, 95%CI: −0.170, 0.654) (Figure 4).

## Discussion

In recent years, whether CPAP therapy can improve brain function and structure has attracted extensive research interest, prompting researchers to study the neuropathological mechanisms of WM alterations in patients with OSA after CPAP therapy. The results showed that, first, extensive changes occurred in WM fiber in patients with OSA before treatment, confirming that WM fiber alterations were related to OSA, mainly involving the ATR, CST, FM, IFO, ILF, UNC, SLF, and CGC. Second, OSA symptoms and cognitive impairment improved 3 months after CPAP treatment in patients with OSA, but WM fiber did not significantly change. The change of WM fiber may be associated with the individual differences in OSA patients and the degree of response to treatment. Third, at baseline, we found that WM fibers were significantly correlated with SaO2 <90%, AHI, and MoCA, indicating that WM alterations were related to cognitive impairment, and intermittent hypoxia was one of the causes of WM alterations.

At baseline, patients with OSA showed extensive WM fiber abnormalities. Long-term intermittent hypoxia is well-known to cause pathological changes such as oxidative stress and ischemia reperfusion in the brain, which leads to WM fiber alteration in the brain (32). WM structural alterations are associated with extensive and chronic cell damage, including decreased synaptic and axon density, neurodegeneration, and cell death (33). Consistent with these findings, we found that the FA values of WM fiber tracts were reduced and RD value increased in patients with OSA, which may be related to the different underlying pathophysiological processes of OSA. Reduced FA is related to the number and density of axons, and increased RD is related to myelin sheath injury (34). The study by Zhang et al. (35) showed that impaired integrity of the anterior corpus callosum fiber bundle is related to early myelination and unique fiber composition, which explains the different susceptibilities of different structures to intermittent hypoxic environments. Mei et al. (36) reported that WM integrity abnormality is only found in children with moderate-to-severe OSA, and WM impairment is accompanied by cognitive decline, confirming that WM fiber impairment is related to OSA severity. Therefore, we hypothesized that OSA severity, age, and physiological and anatomical structures were related to the susceptibility and degree of WM fiber bundle alteration.

The CGC is involved in the control of autonomic nervous functions, including respiration, blood pressure, and salivation (37). During breathing, neurons in the CGC fire in sync with breathing and are associated with the central network that controls breathing (38). Taylor et al. (39) showed that the structural changes in the CGC are related to an increase in sympathetic discharge when awake, and that sympathetic excitatory stimulation can cause changes in the central nervous system and thus participate in the regulation of blood pressure and respiration. The corticospinal tract originates from the motor-related cortical region, mainly carries motor-related information, and mediates voluntary movement (40). Previous studies have shown that impaired peripheral muscle movement in patients with OSA is associated with decreased corticospinal excitability (41). Marillier et al. (42) showed that intermittent hypoxia and hypercapnia during sleep lead to increased corticospinal tract inhibition in patients with OSA, partially explaining the neuromuscular mechanism of OSA limb muscle dysfunction. Similar to previous results, our study further confirmed fiber alteration in the CGC and corticospinal tract. Therefore, we speculate that sleep disruption in patients with OSA may lead to intermittent hypoxia and hypercapnia, which may indirectly affect the autonomic nerves related to respiration and lead to muscle movement disorders, which may be one of the risk factors for exacerbating the progression of OSA.

In addition, we found that WM fiber alterations in ATR, FM, IFO, ILF, UNC, SLF, and other regions. Numerous studies have shown that various cognitive functions, including reading, intelligence, information processing, visuospatial memory, attention, and response inhibition, are associated with different WM microstructures (43, 44). The ATR and SLF are involved in speech production disorders (45). The UNC is involved in bidirectional information transmission between the frontal and parietal cortex (46), and the FM is associated with delayed memory disorders (47), The damage of the ILF is significantly associated with depression (48), and the IFO plays an important role in semantic processing (49). Our results provide more evidence of WM microfiber structural alteration in patients with OSA. At the same time, fiber alterations in these different white matter areas may provide more explanations for the heterogeneity of OSA cognitive impairment and symptoms (50). We also found that extensive WM fiber alteration was significantly correlated with SaO2 <90% time, AHI, and MoCA. This further explained that intermittent hypoxia and severity of OSA led to extensive WM fiber bundle damage and contributed to further understanding of the neuroimaging mechanism of WM fiber bundle integrity affecting cognitive function.

In the current study, 3 months of CPAP therapy did not cause in alteration WM in the brain. Understanding whether CPAP therapy for OSA is likely to stabilize and reverse brain structural changes is important because it can provide clinical guidance and motivate patients to adhere to treatment. Studies on the recovery of brain function and structure after OSA treatment have shown inconsistent results. Canessa et al. (51) demonstrated that increased GM volume in the hippocampus, medial orbitofrontal anterior lobe, and superior frontal gyrus is associated with improvements in visuospatial short-term memory, attention, and executive function after 3 months of treatment, confirming that changes in the GM are associated with cognitive improvement. Contrary to the findings of Donoghue et al. (52) no significant difference was found in the GM between the pretreatment and control groups (52). In a voxel-based morphometry study of patients with OSA after surgical treatment, alterations in some brain WM structures were not significant, but were compensated by changes in other cortical areas (53). A recent DTI study showed that some WM tracts reversibly recover after 3 months of short-term treatment (54). The different results of previous studies may be due to the relatively individual difference and varied responses of different patients to CPAP therapy (12).

## Limitation

This study has several limitations. First, this was a short-term CPAP therapy, and We only used DTI to discuss the alterations of WM structure. DTI technology can only show the alterations of WM non-specifically, and cannot confirm the existence of tiny recovery of nerve cells. Second, we examined the general population, which was dominated by men, whose results cannot be extended. Finally, our sample size was relatively small which may cause potential statistical bias.

## Conclusion

This study was based on the spatial statistics of trajectory to investigate the reversible changes in WM microstructure in the brain before and after short-term CPAP treatment. We observed extensive abnormalities in the WM in patients with severe OSA before treatment, and these areas included the autonomic nerves involved in respiration. No significant reversible WM alteration was found after short-term CPAP therapy. Therefore, further longitudinal studies are warranted to explore the reversible alterations in WM microstructure in the brain after long-term treatment and to provide potential imaging markers for clinical treatment.

## Data availability statement

The original contributions presented in the study are included in the article/supplementary material, further inquiries can be directed to the corresponding author.

## Ethics statement

The study was approved by the Ethics Committee of The First Affiliated Hospital of Nanchang University. The patients/participants provided their written informed consent to participate in this study.

## Author contributions

XL and ZW wrote, reviewed, and revised the manuscript. DP guided and designed the MRI experiment. ZW analyzed DTI data. XL and WD analyzed and discussed the ideas of the paper. XL analyzed the results and wrote the manuscript. LC, TL, LH, HL, LK, YS, PL, WX, and YZ collected DTI data and applied for the ethics approval. All authors contributed to the article and approved the submitted version.

## Funding

This study was supported by the National Natural Science Foundation of China (Grant Nos. 81860307 and 81560285); the Natural Science Foundation Project of Jiangxi, China (Grant Nos. 20202BABL216036, 20181ACB20023, and 20171BAB205070); Education Department Project of Jiangxi provincial, China (Grant Nos. 700544006 and GJJ190133); and Department of Health Project and Jiangxi provincial, China (Grant No. 20181039).

## Conflict of interest

The authors declare that the research was conducted in the absence of any commercial or financial relationships that could be construed as a potential conflict of interest.

## Publisher's note

All claims expressed in this article are solely those of the authors and do not necessarily represent those of their affiliated organizations, or those of the publisher, the editors and the reviewers. Any product that may be evaluated in this article, or claim that may be made by its manufacturer, is not guaranteed or endorsed by the publisher.
